# Granulomatous interstitial polymyositis and intramuscular neuritis in a dog

**DOI:** 10.1186/s13028-021-00579-x

**Published:** 2021-03-30

**Authors:** Josefin Hultman, Marco Rosati, Tone Kristensen Grøn, Kaspar Matiasek, Cathrine Trangerud, Karin Hultin Jäderlund

**Affiliations:** 1 Department of Companion Animal Clinical Sciences, Faculty of Veterinary Medicine, Norwegian University of Life Sciences, Ullevålsveien 72, 0454 Oslo, Norway; 2 Present Address: Department of Companion Animal Clinical Sciences, Faculty of Veterinary Medicine, Norwegian University of Life Sciences NMBU, Postboks 5003, 1432 Ås, Norway; 3 Section of Clinical & Comparative Neuropathology, Centre for Clinical Veterinary Medicine, Ludwig-Maximilians-Universität, Veterinärstr. 13, 80539 Munich, Germany

**Keywords:** Auto-immune, Canine, Idiopathic, Immune-mediated, Myositis, Sarcoidosis

## Abstract

**Background:**

Granulomatous myositis is a rare condition in both humans and dogs. In humans it is most frequently related to sarcoidosis, where a concurrent granulomatous neuritis has been reported occasionally. Simultaneous granulomatous myositis and neuritis have been diagnosed previously in dogs (unpublished observations), but have not been studied further. Additional investigations are therefore warranted to characterize this disorder. Here we present a detailed description of concurrent idiopathic granulomatous myositis and granulomatous neuritis in a dog with suspected immune-mediated aetiology.

**Case presentation:**

The dog presented with dysphonia and paresis in the pelvic limbs and tail. In addition to muscle biopsies being taken for histopathology, magnetic resonance imaging, computed tomography and electrodiagnostics were performed. Muscle biopsies displayed granuloma formation with giant cells and epithelioid macrophages in muscle fibres and nerve branches. Microorganisms were not detected. Long-term treatment with glucocorticoids was clinically successful. Two years after the clinical signs started, the dog presented with signs of sepsis and died. Histopathologically, no granulomatous inflammation could be demonstrated in either muscles or nerves at that time.

**Conclusions:**

This case illustrates a granulomatous interstitial polymyositis and intramuscular neuritis that improved clinically and resolved histologically with glucocorticoid treatment. Idiopathic granulomatous myositis and neuritis should be considered as a differential diagnosis in dogs with clinical signs of neuromuscular disorders.

## Background

Granulomatous inflammation is a specific form of chronic inflammation predominantly characterized by mononuclear leukocytes, especially histiocytes, but also epithelioid cells and multinucleated giant cells [1]. Granulomatous myositis is an infrequent disorder in humans and is usually associated with sarcoidosis [2], a systemic multi-organ disease of uncertain aetiology. Sarcoidosis may also manifest as granulomatous neuritis [3]. Cases of granulomatous myositis occur sporadically in dogs, but are rarely reported. In a review of 200 dogs with inflammatory myopathy, seven dogs fell outside the traditional classification groups. Histologically, the lesions found in those seven dogs were characterised by either collagen-vascular diseases, granulomatous myositis, or sarcoid-like myopathy [4]. However, the authors emphasised that further investigations were needed to classify those disorders more accurately. Studies of a simultaneous granulomatous neuritis in dogs seem to be non-existent. The present case report is a detailed description of concurrent idiopathic granulomatous interstitial polymyositis and intramuscular neuritis in a dog, which was successfully treated with glucocorticoids.

## Case presentation

A 6-year-old, Norwegian born, intact male Russian Tsvetnaya Bolonka, 6 kg, was referred to the Norwegian University of Life Sciences (NMBU) at the Small Animal Teaching Hospital with a 4.5-months history of progressive weakness. Starting with weakness of the tail and reduced general condition, the clinical signs progressed to include dysphonia, muscle pain, and lameness of the pelvic limbs, more prominent on the right. Two months prior to referral, magnetic resonance imaging (MRI) and computed tomography (CT) of the lumbar region had been performed, demonstrating asymmetrical pelvic limb muscle atrophy involving particularly the right iliopsoas and quadriceps musculature. Several years before the first clinical signs of the progressive weakness appeared the dog was diagnosed with hypothyroidism and had been treated with substitution therapy since then. He had never left Norway.

General physical examination revealed reduced muscle mass of the proximal muscles of the right pelvic limb and the dog was afebrile (38.8 °C). On neurologic examination, the dog was ambulatory but paraparetic, more pronounced on the right, with an obvious hypoflexion of the hip joint, bunny-hopping gait, and a paretic tail. Cranial nerves were normal except for the observed dysphonia, whereas postural reactions were decreased in both pelvic limbs with reduced spinal reflexes in the right pelvic limb. The neuroanatomic localisation was considered to be the neuromuscular system. Differential diagnoses included inflammatory, as well as non-inflammatory, myopathies/neuropathies.

A complete blood count (CBC) and serum biochemistry profile revealed mildly increased creatinine phosphokinase, but was otherwise unremarkable. *Toxoplasma gondii*-specific IgM titre was border line (1:32) while IgG was negative, as was a follow-up IgM titre. *Neospora caninum* antibody titres were negative, and serum total thyroxine concentration was within the reference range.

**Fig. 1:**
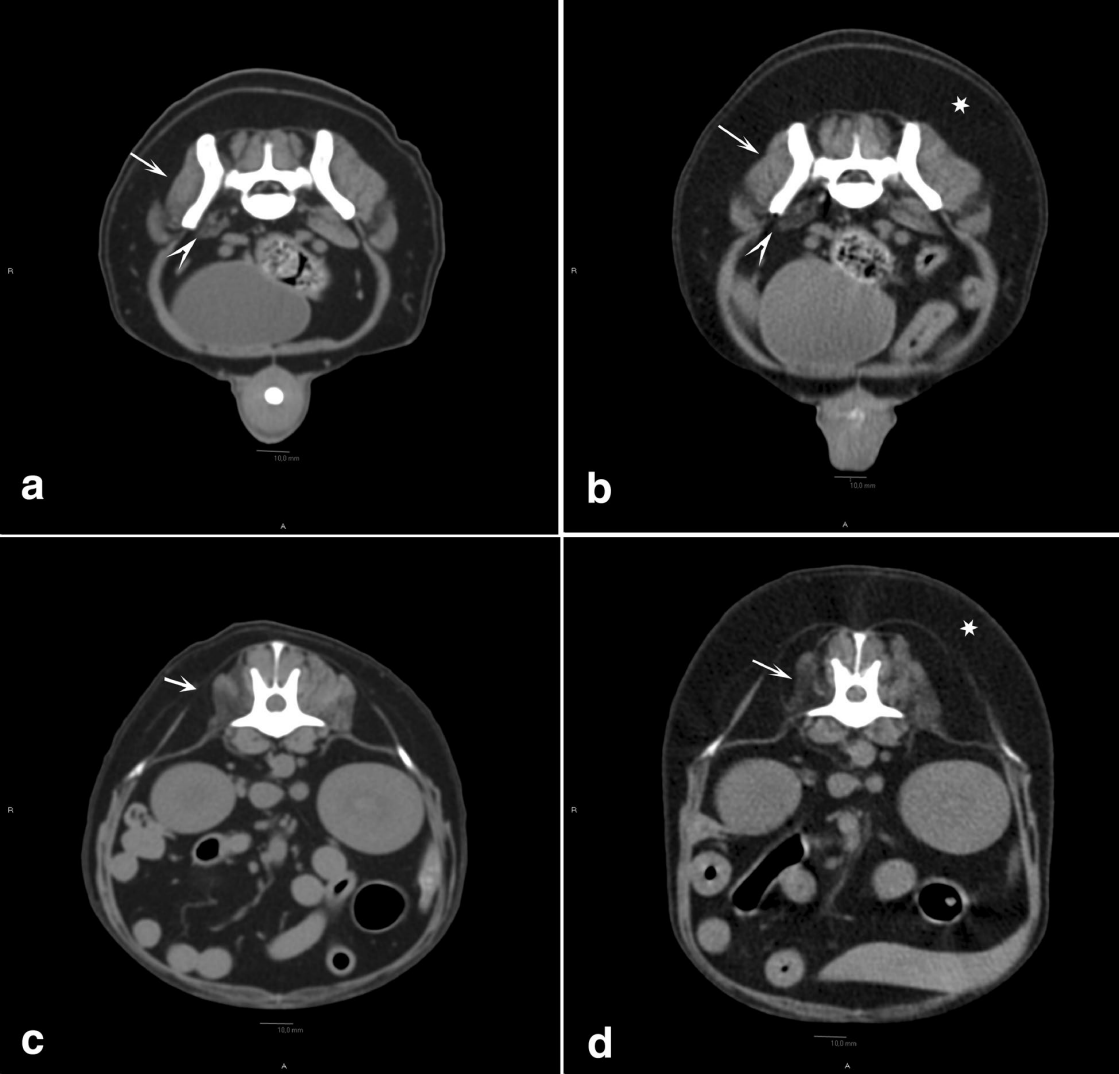
Computed tomographic images pre- and post-prednisolone treatment in a dog with granulomatous myositis and neuritis. Transverse computed tomographic (CT) images using a soft tissue algorithm, prior to prednisolone treatment (**a**, **c**) and at a revisit 17 months later (**b**, **d**). Transverse CT images at the level of the caudal aspect of L7 (**a**, **b**) and at the level of the intervertebral space between L1 and L2 (**c**, **d**). Atrophy of the right medial gluteal muscle (arrow) and iliopsoas (arrowhead) (**a**, **b**). Atrophy of the right longissimus thoracic, longissimus lumborum, multifidus lumborum and iliocostal lumborum muscles (arrow) (**c**, **d**). The muscle atrophy appears unchanged when comparing the two studies. There is moderately to markedly increased body habitus, predominantly subcutaneous fat (asterisk), at the revisit (**b**, **d**) compared to the first study (**a**, **c**)

**Fig. 2:**
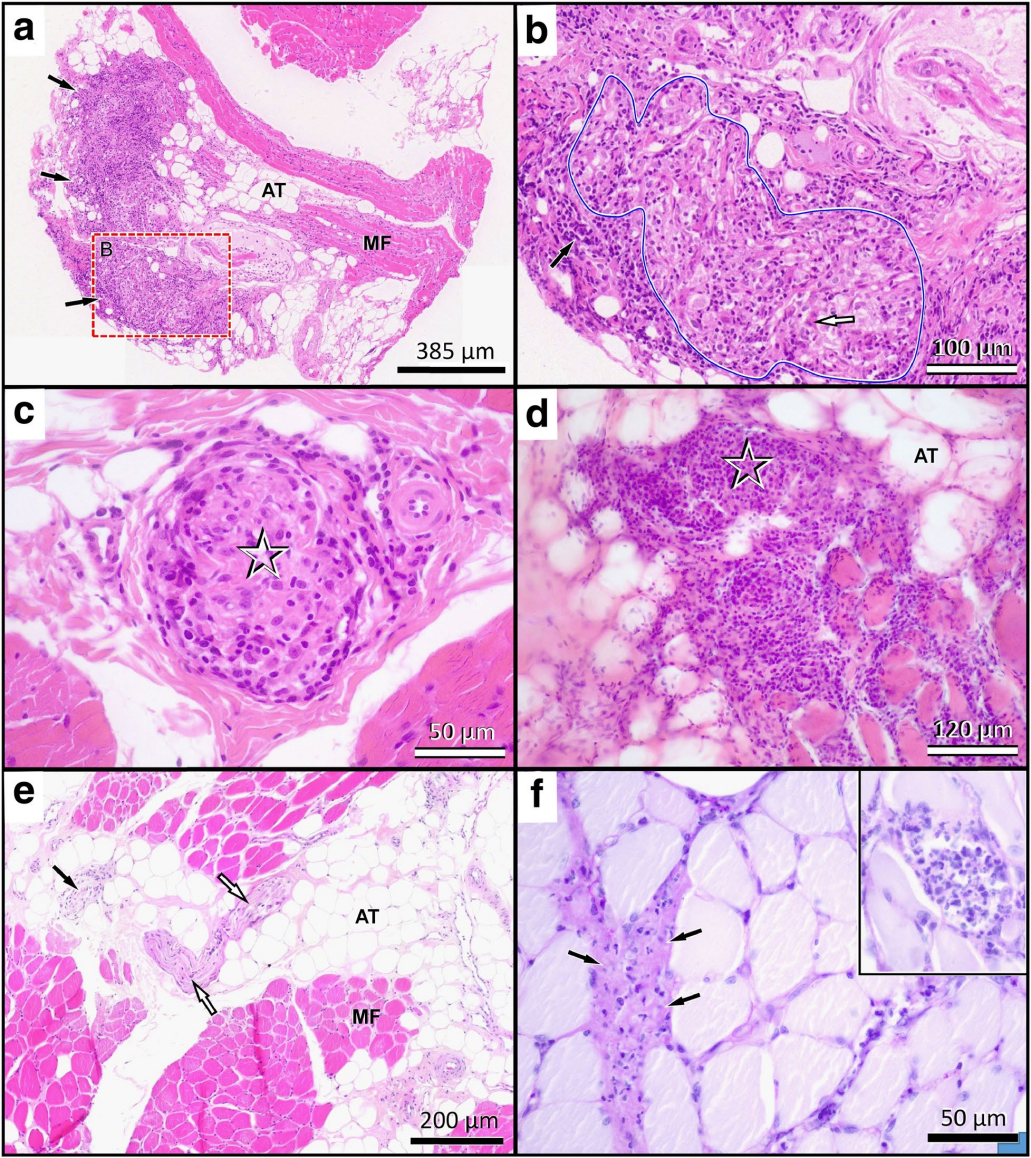
Representative images of histological muscle changes at time of *intravitam* diagnosis and 2 years later. **a** At initial presentation muscle biopsies showed proliferative inflammatory changes (black arrows) within expanded interstitial tissue, sparse myofiber (MF) density and adipose replacement tissue (AT; “fatty” replacement). **b** At higher magnification of the framed area in **a**, intramuscular nerve branches (blue lined) are severely enlarged and their fascicular architecture is effaced by granulomatous changes with macrophages and multiple multinucleated giant cells (white arrow). Further mixed leukocytic infiltrates with lymphocytes, plasma cells and scattered neutrophils are also evident in the epineurial perimysium (black arrow). **c** The changes extend along most intramuscular nerve twigs (asterisk) even if perifascicular changes are sparse. **d** In other areas, as seen in this cryosection, inflammatory infiltrates affect the endomysial branches (asterisk) and from there invade deeply in between the myofibres. **e** No such granulomatous lesions are seen at post mortem examination 2 years later while the adipose tissue (AT) has grown. Some nerve branches show myelinated fibre loss and endoneurial fibrosis (black arrow), while others present with normal histological appearance (white arrows). **f** Many fascicles however show necrofibrinopurulent interstitial changes (black arrows) as well as scattered necrotic myofibres infiltrated by hypersegmented polymorphonuclear neutrophils (inlet). Both phenomena are compatible with sepsis. Slide technique and stains: **a**–**c**, **e** Formalin fixation/paraffin embedding, haematoxylin–eosin; **d** cryosection, haematoxylin–eosin; **f** Formalin fixation/paraffin embedding, periodic acid Schiff.

By the time of a planned revisit to NMBU for laryngeal inspection, diagnostic imaging, electrodiagnostics, and biopsies 2 weeks after referral, the clinical signs had progressed further and the dog was now showing general paresis in all four limbs. During a light plane of anaesthesia, laryngeal inspection was performed showing immobile arytenoids and vocal cords bilaterally, indicative of a laryngeal paresis causing the dysphonia. Pre- and post-contrast CT examinations of the whole body were performed under general anaesthesia in dorsal recumbency. Iohexol (Omnipaque 300 mgI/mL, GE Healthcare AS, Oslo, Norway) dosed at 600 mg iodine/kg was administrated intravenously and CT images were acquired using a 4-detector row CT scanner (BrightSpeed, GE Healthcare), 1.25 mm slice thickness, 0.625 mm overlap, using helical acquisition in bone and soft tissue algorithms. The CT-examination revealed bilateral muscle atrophy with various degrees of fat infiltration evident in the muscles of the thigh, thoracic limbs, and head. The muscle atrophy was most prominent on the right side of the back (Fig. 1), right pelvic limb, and right aspect of the head (temporal and masseter muscles). Changes in the triceps musculature were most prominent on the left side. Electromyography performed under general anaesthesia showed spontaneous activity in multiple muscle groups. Motor nerve conduction velocity was measured to be 40.5 m per second (m/s) (mean ± SEM in healthy dogs: 60 ± 1.1 m/s [5]) in the ulnar nerve, and no other nerves were tested. Muscle biopsies were taken from the right biceps femoris muscle, the left triceps muscle, and the longissimus dorsi muscles bilaterally, and submitted for a routine muscle biopsy panel established at the Neuropathology Laboratory, Ludwig-Maximilians-Universität, München, Germany. On histological examination, all muscle samples revealed multifocal interstitial and occasionally fibre-directed lymphohistiocytic infiltrates which, in some fields, were accompanied by granulomatous changes with giant cells and/or epithelioid macrophages (Fig. 2). Intramuscular nerve branches were diffusely affected by endoneurial granulomatous changes. Perimysium and endomysium showed moderately increased amounts of fibrocollagenous tissue accompanied by patchy areas of mild perimysial lipomatosis. Grocott’s methenamine silver stain for fungi, Fite Faraco stain for acid-fast bacteria and Gram stain were all negative. Likewise, 16s rRNA polymerase chain reaction was negative for bacterial sequences. Based on these findings, aseptic granulomatous polymyositis and neuritis were diagnosed.

Following the negative microbial investigations, treatment was initiated with glucocorticoids (prednisolone) 1.7 mg/kg per os (p.o) twice daily for 2 weeks, followed by gradual tapering to a lower maintenance dose. At a revisit 1 month later, clinical signs had improved but were still present. Gradual improvement of the clinical signs continued over the next few months and then stabilised. At this point, neurologic examination revealed mild reductions of the right pelvic limb flexor reflex and the patellar reflex. Gait evaluation showed circumduction of the right pelvic limb but was otherwise normal. There were no signs of dysphonia or laryngeal paralysis and the tail had regained its normal position. Approximately 1 year after initiating treatment the dog had a mild relapse of clinical signs while receiving prednisolone at a dose of 0.75 mg/kg every third day. Following a dose increase, the owner reported an immediate positive response. The dog was re-examined again 16 months after initiating treatment. Gait evaluation was normal except for a mild gait disturbance of the right pelvic limb. Electromyography was repeated and spontaneous activity was still observed in several muscle groups, but to a lesser extent than before. Motor nerve conduction velocity of the right ulnar nerve was measured to be 48.0 m/s. A CT was performed and revealed similar findings as previously, except for an increased amount of subcutaneous fat, predominantly of the dorsum, considered most likely to represent an adverse effect of prednisolone treatment.

Two years after the first visit, the dog presented to the Emergency Service at NMBU with clinical signs of lethargy, pyrexia (40.2 °C), weakness, and coughing. Blood analysis, ultrasound, and CT were in agreement with a septic condition and the dog was treated accordingly. However, three days later the dog went into cardiac arrest and died. Autopsy was performed at the pathology department of NMBU and further muscle/nerve samples were sent to the Neuropathology Laboratory, Ludwig-Maximilians-Universität, München, for follow-up. The autopsy revealed purulent myocarditis, fibrinous pericarditis, interstitial nephritis, and centrilobular necrosis and thrombosis of the liver. Several muscle samples showed changes corresponding to a multifocal, subacute suppurative myositis with fibre necrosis. Muscle atrophy and intermediate neuropathy featuring axonal atrophy and partial demyelination were also present. No granulomatous changes were seen at that stage. Adipose tissue in between the muscle fibres had increased, which is expected when inflammatory changes is restricted to the interstitial compartments (Fig. 2). Microbiology samples taken from the liver and spleen showed moderate growth of *Escherichia coli,* confirming the final septic condition.

## Discussion and conclusions

This case report describes a dog with histopathologic changes in several muscles suggestive of idiopathic granulomatous myositis with concurrent granulomatous neuritis. This condition has not previously been reported in canids. Granulomatous myositis has been described in single case reports of dogs infected by *T. gondii* [6], *N. caninum* [6], *Trypanosoma cruzi* [7], *Sarcocystis* spp. [8], *Leishmania infantum* [9]; as well as associated with thymoma [10], lymphoma [11] and non-infectious disease of unknown aetiology [4]. Granulomatous neuritis may potentially be associated with *N. caninum* infection in dogs [12].

Besides *N. caninum* [13] and unspecified *Toxoplasma* spp. [14] none of the infectious causes of canine granulomatous myositis and neuritis have been reported as endemic infections in dogs in Norway. *Sarcocystis* spp. infection, with dogs as definitive host, is however sporadically seen in dogs in Norway (B. Gjerde, personal communication). In our case, no signs of any underlying infectious disease causing the clinical signs was found. An infectious aetiology was therefore considered unlikely in this case. The fact that the dog responded to glucocorticoid treatment and remained well over a 2-years period also makes an immune-mediated disease more probable. Moreover, no evidence of other conditions associated with granulomatous myositis, such as thymoma or lymphoma, was diagnosed. Hence, no underlying cause was found for the granulomatous myositis and neuritis in this case.

In humans, granulomatous myositis and neuritis can be related to various infectious and non-infectious diseases. The most frequent cause for granulomatous myositis with granuloma formation in humans is sarcoidosis [15], which may simultaneously cause granulomatous neuritis. In symptomatic cases of the muscular form, paresis and muscle atrophy are the most prominent clinical signs [16]. Magnetic resonance imaging and CT may reveal muscle atrophy, electromyography usually demonstrates abnormal activity in affected muscles [16] and, with concomitant peripheral nerve involvement, slower nerve conduction velocities can be registered [17]. Many features of the muscular and neurological form of the human sarcoidosis resemble those seen in this patient. Since this dog’s clinical signs were limited to the neuromuscular system, the diagnostic work-up focused on that area and we cannot exclude that other organ systems were affected. However, a whole-body CT, CBC, and serum biochemistry profile did not reveal any suspicious abnormalities. Granulomatosis with polyangiitis (Wegener’s) is a human disease of unknown aetiology that is characterized by vasculitis and necrotizing granulomatous changes, affecting primarily the respiratory system and kidneys. Peripheral nerve involvement has been reported in up to one third of cases. Muscle involvement is rare and biopsies of nerves display axonal degeneration [18]. Neither necrotizing granulomas nor vasculitis were described in our case.

Recommended treatment for sarcoidosis in humans is long-term glucocorticoids or other immunosuppressants [19]. The response to glucocorticoids for muscular sarcoidosis is unpredictable [15, 20], whereas patients with peripheral nerve involvement seem to respond well to this treatment [21]. In our case, a dramatic improvement in clinical signs was seen shortly after initiation of glucocorticoids, although the signs were not completely abolished. Due to a relapse of clinical signs 1 year after diagnosis, the dose of glucocorticoids was increased resulting in an immediate response, which has also been described in humans with relapse of neurosarcoidosis [22]. Two years after diagnosis our patient was still on prednisolone (0.8 mg/kg p.o every other day) and presented to our emergency service with sepsis and died. Muscle and nerve biopsies were examined post mortem. Nerve samples displayed changes consistent with mild axonal atrophy and partially demyelinating neuropathy, which could be consistent with long-term corticosteroid treatment [23]. No granulomatous changes were observed in nerve fibres, muscles tissue, or other organs, indicating a good response to the glucocorticoid treatment. However, suppurative myositis was diagnosed, probably explained by the septic condition. There is an increased risk for infections when treating with long-term immunosuppressive medications such as corticosteroids [24]. Thus, long-term prednisolone treatment could have been a contributing factor to the septic condition in our patient.

This patient had been diagnosed with hypothyroidism several years earlier. Studies have demonstrated that human patients with sarcoidosis are more commonly suffering from thyroid autoimmunity when compared with the general population [25]. This indicates a possible unknown association between the two different diseases. If also applicable to dogs, the idiopathic granulomatous neuritis and myositis may have been a part of a general predisposition to autoimmune disorders in this patient.

In conclusion, this case report describes a dog with idiopathic granulomatous interstitial polymyositis and intramuscular neuritis that resemble the muscular form of the human disease sarcoidosis. Our patient responded well to glucocorticoid treatment, and histopathologic examination of repeated muscle and nerve biopsies could not reveal any granulomatous changes in the specimens after two years of treatment.

## Data Availability

The datasets generated during and/or analysed during the current study are not publicly available due to the integrity of the clinical record and the privacy law covering both the dog and dog owner. The datasets could, however, be available for the editor from the corresponding author upon request.

## Abbreviations

CBC: Complete blood count
CT: Computed tomography
MRI: Magnetic resonance imaging
NMBU: Norwegian University of Life Sciences
SEM: Standard error of mean
